# Effect and safety posterior scleral reinforcement on controlling myopia in children: a meta-analysis

**DOI:** 10.1007/s10792-024-02929-w

**Published:** 2024-02-06

**Authors:** Jing Chen, Yu Tang, Zhihong Lin, Zhengyang Tao, Hongwei Deng

**Affiliations:** 1 Shenzhen Eye Hospital, Shenzhen Eye Institute, Jinan University, No. 18 Zetian Road, Futian District, Shenzhen, 518040 Guangdong China; 2 The Second Clinical Medical College, Jinan University, Shenzhen, China

**Keywords:** Posterior scleral reinforcement, Myopia, Children, Effect, Meta-analysis

## Abstract

**Purpose:**

The aim of this meta-analysis was to assess the efficacy of posterior scleral reinforcement (PSR) on the control of pediatric myopia. Electronic databases were systematically searched.

**Methods:**

Standardized mean differences (SMDs) of outcomes were calculated. Eight studies with 357 patients with pediatric myopia were included. The SMD for the increase in mean axial length (AL) in the PSR and control group was − 1.19 (95% CI − 1.71, − 0.68).

**Results:**

The SMD for decrease of best-corrected visual acuity (BCVA) LogMAR in the PSR and control group was 0.85 (95% CI 0.28, 1.43). The SMD for change in intraocular pressure (IOP) at the time of surgery and at the end of the follow-up period in the PSR group was − 0.01 (95% CI − 0.48, 0.47).

**Conclusion:**

This meta-analysis indicates that PSR surgery may be an effective therapeutic strategy to control the progression of myopia in childhood with acceptable adverse effects.

**Supplementary Information:**

The online version contains supplementary material available at 10.1007/s10792-024-02929-w.

## Introduction

Myopia has become a major global public health problem [1, 2], especially high myopia, because it is progressive, its complications can lead to visual impairment, low vision, and even blindness, thus it has attracted great attention [3]. Visual acuity decline is caused by myopic macular degeneration, which is strongly related to the length of ocular axis [4–6]. Myopia is prevalent in East and Southeast Asia. The morbidity of myopia among young people is about 80–90%, accompanied by a relatively high prevalence of high myopia among young people (10–20%) [7]. It is predicted that by the year of 2050 there will be 4.8 billion myopic people (nearly half of the world population) and 938 million people with high myopia (10% of the global population) [8]. Studies have shown that when children develop myopia at a lower age, myopia progresses faster [9, 10]. Therefore, the preventing or control of the progression of myopia in myopic children is of significant importance [11].

Extensive high myopia includes persistent lengthening of the eyeball, thinning of the sclera and localized expansion of the posterior sclera [12, 13]. It is worth noting that the axial length is closely related to refractive status, because long eyes are more inclined to be nearsighted than short eyes [14]. Therefore, the control of the elongation of ocular axis throughout childhood may be very important to keep normal vision (at least 20/20 using a Snellen chart) and may become the main goal of preventing myopia [15, 16]. Posterior scleral reinforcement (PSR) was first proposed by Shevelev and later improved by Thompson [14, 17]. The use of posterior buckles on the thinner sclera can provide local stabilization, which may slow down the development of axial elongation and myopia [18]. Although the surgical effectiveness may be controversial, several studies have confirmed the benefits of stabilizing axial lengthening and refractive status [19–21]. Nevertheless, this approach of surgery remains to be performed at considerable ophthalmic centers in a great number of countries for patients with myopia including children [14, 21–27].

No systematic review and meta-analysis have yet been performed to investigate or quantitatively define the therapeutic effect of PSR surgery in children. The aim of this study was to quantify the efficacy of PSR on the control of axial elongation and refractive progression via meta-analysis.

## Materials and methods

The meta-analysis was conducted based on the guidance of the updated Preferred Reporting Items for Systematic Reviews and Meta-analysis (PRISMA) [28]. All included studies had declared ethical approvals and informed consent in the original articles, no ethical approval or informed consent was needed in this study.

### Search strategy and study selection

We comprehensively searched the electronic databases including Pubmed, Embase, Web of science and the Cochrane Library up to June 23, 2022. Only citations in English were included. The following keywords and terms were used: myopia, scleral buckle, posterior scleral reinforcement, buckling, reinforcement, scleroplasty, snyder thompson. Search strategies for databases was provided in Table S1**.** In addition, the references of the included articles were also screened for additionally eligible studies. Inclusion criteria were as follows: (1) ≤ 18 years old (2) myopia; (3) PSR operation was used for controlling myopia progression; (4) Studies evaluated the indicators for surgery effects and/or safety such as mean change in spherical equivalent (SE) from the time of surgery to the end of the follow-up period in the PSR and control group, the increase in mean axial length (AL) in the PSR and control group, decrease of best-corrected visual acuity (BCVA) LogMAR in the PSR and control group, change of axial length/horizontal corneal radius of curvature ratio (AL/hCRC) in the PSR and control group, and change in intraocular pressure (IOP) at the time of surgery and at the end of the follow-up period in the PSR group (the decrease of SE, AL, AL/hCRC, IOP while the increase of BCVA represent the alleviation of myopia). Exclusion criteria included: (1) The data to be analyzed cannot be extracted or calculated; (2) Case reports or series, reviews, comments, editorials, and animal studies; (3) Non-English reports. If studies were performed by the identical research group, studies with the most sufficient information were included. Two independent researchers performed the database search and study selection. Disagreements were addressed through discussion.

### Data extraction and quality assessments

Two investigators (Yu tang & Jing Chen) independently screened the title and abstract of enrolled articles on the basis of the inclusion criteria. Then a full-text evaluation of the studies was performed for the final eligibility. Moreover, the following information of included studies was extracted or calculated from the raw data provided in the articles included: family name of the first author, year of publication, country of participants, number of participants, and operation outcomes aforementioned. The Newcastle Ottawa Scale (NOS) for cohort studies was used to judge the risk of bias for each included study.

### Statistical analysis

The R Project for Statistical Computing (Version 4.0.2) was used for statistical analyzes on the study level. We calculated pooled estimates of the standardized mean difference (SMD) of outcomes aforementioned in both PSR and control groups, with their respective 95% confidence intervals (CIs). The Cochran *Q* and *I*^2^ statistics were introduced to qualitatively and quantitatively explore the heterogeneity in studies included. Nonsignificant, low, moderate, and high heterogeneity were rated as *I*^2^ values of 0%-, 25%-, 50%, and 75%-, respectively [29]. *Egger’s* method was used to statistically test publication bias [30]. Sensitivity analysis was used to evaluate the robust of the pooled overall outcomes. A *p* value < 0.05 was considered to be statistically significant [31].

## Results

### Study selection and characteristics

**Fig. 1:**
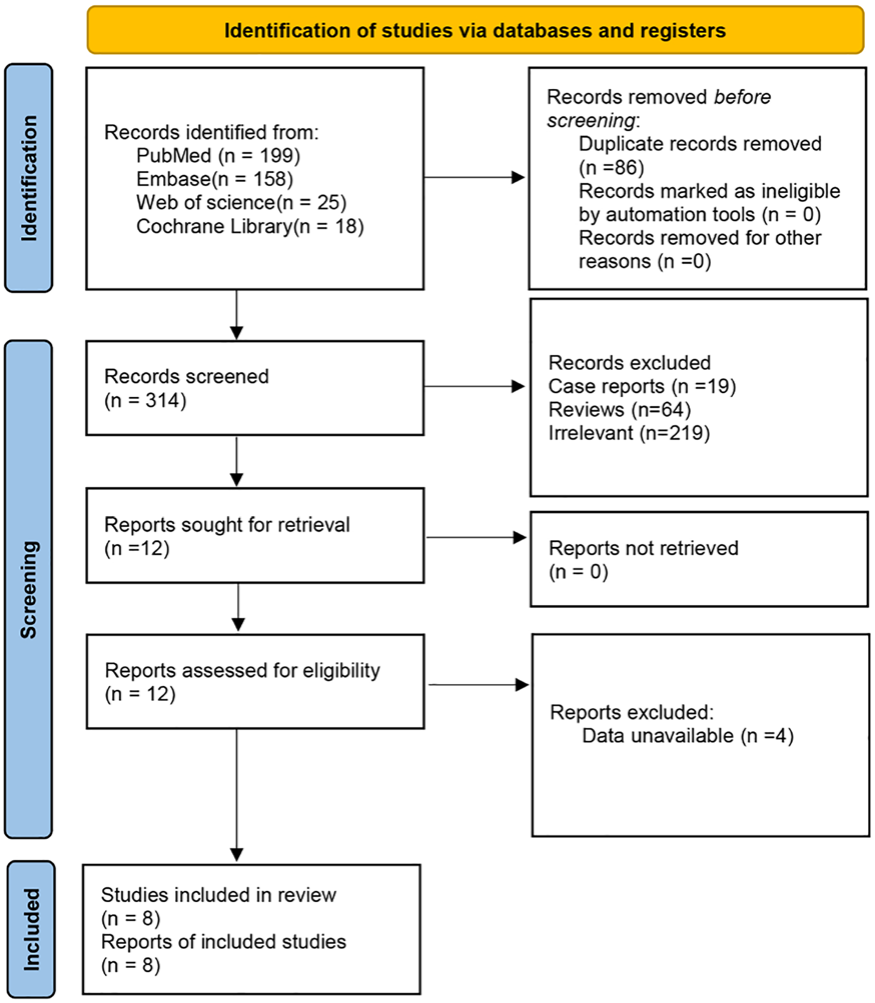
Flowchart of the literature search

Four hundred citations were identified from the databases searched. Eighty-six duplicates were removed and 302 studies were excluded through an initial screening. After a full text reading of the remaining 12 articles, 8 studies comprising 357 patients were finally regarded as inclusion in this meta-analysis (Fig. 1). Additional citations were not identified through bibliography screening of the enrolled articles. Baseline characteristics of included studies were displayed in Table 1. The quality of included studies was assessed as high in accordance with the Newcastle Ottawa Scale (Table S2).

**Table 1:** Study characteristics

Name of first author	Year of publication	Country	Study design	Number of participants	Age, yrs	Intervention	Control
Chen	2013	China	Nonrandomized controlled Cohort	52	8.5 (2.4–16.4)	Surgical procedure was a modified Snyder–Thompson PSR using a strip of homologous duramater, 6 cm long and 0.8 cm wide	children with natural progression of high myopia
Dong	2020	China	Nonrandomized controlled Cohort	89	7.3 (3–17)	Snyder–Thompson posterior scleral reinforcement with round scleral patches	children with natural progression of high myopia
Gao	2022	China	Single arm cohrot	19	4.9 (2–10)	The modified Snyder–Thompson type posterior sclera reinforcement surgery	Self control
Hu	2018	China	Nonrandomized controlled Cohort	67	NR	A buckle of bovine pericardium with a width of 6 to 10 mm was placed onto the sclera surface of the macula in pathological myopic eyes following the Thompson technique	pathologic myopic eyes with posterior staphyloma (23 patients) untreated
Szell	2021	Hungary	Nonrandomized controlled Cohort	52	11.5 (6–18)	The Snyder–Thompson simplified, single-band method was applied under general anesthesia, and halves of a 10 mm wide (5 mm) lyophilized human fascia lata band (Tutogen gmbh, Neunkirchen am Brand, Germany) were implanted to reinforce the posterior pole sclera	Age and myopia-matched subjects (whose parents refused surgery)
Xue	2014	China	Cohort	30	7.5 (4–15)	Only one eye of each patient had posterior scleral reinforcement surgery. The reinforcement scleral flap (40 by 11 by 0.8 mm) was passed underneath the inferior oblique, external rectus, and inferior rectus sequentially with the help of the traction sutures and a muscle hook	Fellow eye
Xue	2018	China	Cohort	40	10 (3–17)	PSCR with a genipin-crosslinked donor scleral strip. Under general anesthesia a 210° conjunctival peritomy was performed along the inferior temporal limbus followed by the exposing and isolation of the inferior and lateral rectus muscles. Traction sutures were placed around the two ocular muscles to pull the anterior side of the globe toward the superior nasal side	Fellow eye
Zhu	2014	China	Cohort	8	12.8 (8–17)	Only one eye of each patient had posterior scleral reinforcement surgery. The procedure of PSR was identical with that used in the study of Xue et al. [26]	Fellow eye

### Treatment effects

**Fig. 2:**
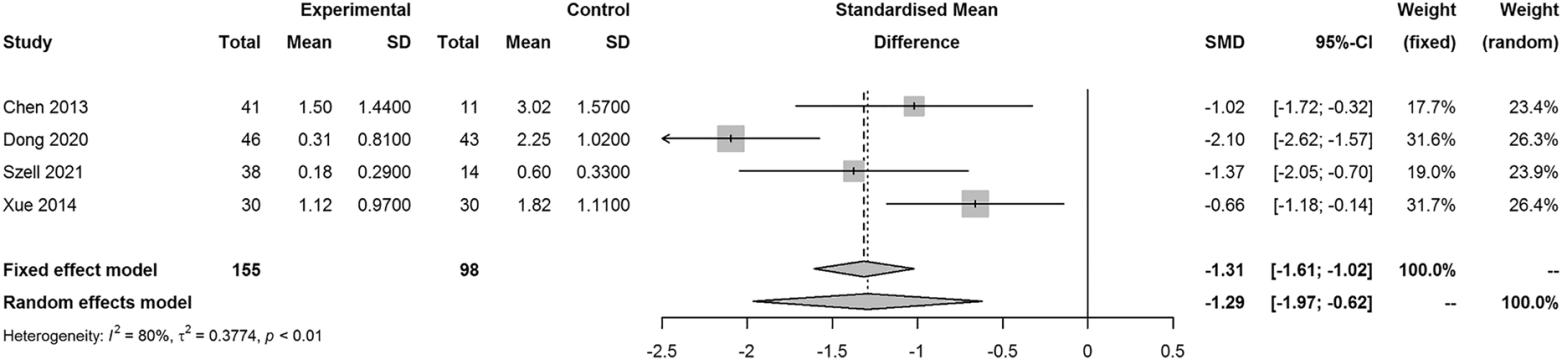
Forest plot of SMD for mean change in SE from the time of surgery to the end of the follow-up period in the PSR and control group

**Fig. 3:**
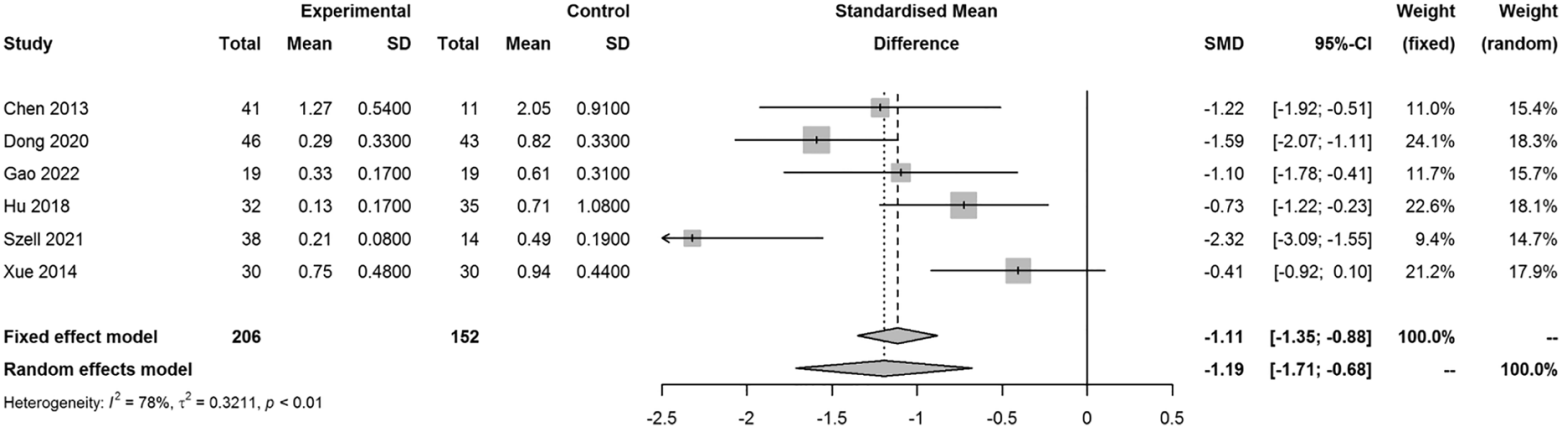
Forest plot of SMD for the increase in AL in the PSR and control group

Four studies reported outcomes on SE, the SMD for mean change of SE from the time of surgery to the end of the follow-up period in the PSR and control group was − 1.29 (95% CI − 1.97, − 0.62; *I*^2^ = 80%, *p* < 0.01), which indicated better performance of PSR than the control group (Fig. 2). Six included studies reported results on AL, the SMD for the increase of AL in the PSR and control group was − 1.19 (95% CI − 1.71, − 0.68; *I*^2^ = 78%, *p* < 0.01), which indicated better performance of PSR than the control group (Fig. 3).

**Fig. 4:**
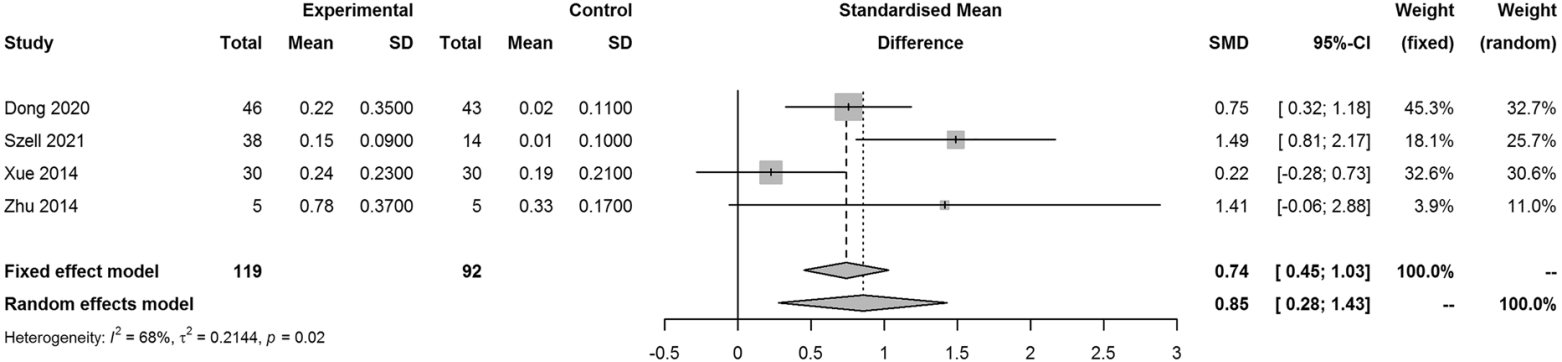
Forest plot of SMD for decrease of BCVA in LogMAR in the PSR and control group

**Fig. 5:**
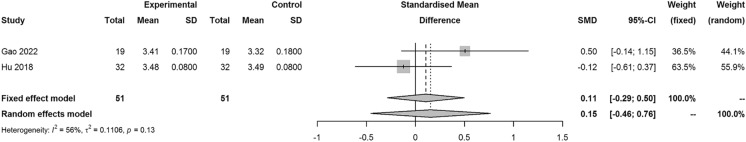
Forest plot of SMD for the mean change of AL/hCRC in the PSR and control group

Four studies reported outcomes on BCVA, the SMD for increase of BCVA LogMAR in the PSR and control group was 0.85 (95% CI 0.28, 1.43; *I*^2^ = 68%, *p* = 0.02), which indicated better performance of PSR than the control group (Fig. 4). Two studies reported results on AL/hCRC, the SMD for increase of AL/hCRC in the PSR and control group was 0.15 (95% CI − 0.46, − 0.76; *I*^2^ = 56%, *p* = 0.13), which indicated better performance of the control group than PSR (Fig. 5).

### Safety

**Fig. 6:**
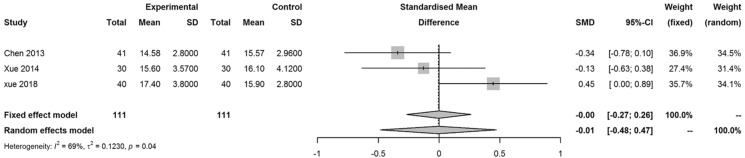
Forest plot of SMD for change in (IOP) at the time of surgery and at the end of the follow-up period in the PSR group

Conjunctival congestion and edema were the most common adverse events among the patients who underwent PSR in included studies and alleviated after several weeks. Three studies reported outcomes on IOP, the SMD for increase of IOP at the time of surgery and at the end of the follow-up period in the PSR group was − 0.01 (95% CI − 0.48, 0.47; *I*^2^ = 69%, *p* = 0.04), which revealed the decrease of IOP was greater in PSR group than the control group (Fig. 6).

### Publication bias

Significant publication bias was not detected by *Egger’s* tests for all aspects of the analysis. The respective *p* values were 0.8443, 0.3516, 0.9260, and 0.4882 for the pooled analysis of SE, AL, BCVA and IOP (Figures S1–S4).

### Sensitivity analysis

Results of the sensitivity analysis indicated that no outlier was identified in all sensitivity analysis (Table 2).

**Table 2:** Quality evaluation of included studies

Study	Representativeness of the exposed cohort	Selection of the non-exposed cohort	Ascertainment of exposure	Demonstration that the outcome of interest was not present at the start of the study	Comparability of cohorts on the basis of the design or analysis	Assessment of outcome	Was follow-up long enough for outcomes to occur	Adequacy of follow up of cohorts	Total quality scores
Chen [14]	1	0	1	1	1	1	1	1	7
Dong [22]	1	1	1	1	1	1	1	1	8
Gao [23]	1	0	1	1	1	1	1	1	7
Hu [24]	1	1	1	1	1	1	1	1	8
Szell 2021	1	0	1	1	1	1	1	1	7
Xue [26]	1	1	1	1	1	1	1	1	8
Xue [27]	1	1	1	1	1	1	1	1	8
Zhu [28]	1	1	1	1	1	1	1	1	8

The maximum score on the NOS is 9 (highest quality), and we assigned scores of 0–3, 4–6, and 7–9 for low, moderate, and high quality of studies, respectively

## Discussion

Myopia poses a great impact on public health and socio-economic well-being [8, 32, 33]. Myopia refers to the increase of axial length and the thinning of sclera, which may be due to the decrease of collagen synthesis and the increase of collagen degradation [34–36]. Generalized high myopia may involve scleral degeneration, eye lengthening, and choroidal, macular, and peripheral retinal lesions [37]. Posterior sclera reinforcement can control the development of myopia. This meta-analysis looked at PSR’s effects in treating myopia in a pediatric population. It concluded that the PSR group showed significant improvements in SE, AL, and BCVA compared to the control group, with no significant adverse effects.

Previous laboratory studies have shown that the strengthening of the sclera to the fovea can improve the microcirculation in the macula [35, 38]. Furthermore, current data support that PSR operation imposes eye elongation and show advantages of this surgery. Although the IOP change in PSR group was statistically significant from preoperative to the end of follow-up, we believe that this change is not clinically significant. Furthermore, slight edema in conjunctiva and congestion were commonly found in the PSR group in several included studies and were alleviated over several weeks. Due to limited information on adverse event rates, results on these rates were not synthesized. In addition, the Snyder–Thompson surgery type were used in most included studies, the surgical procedure utilized in the Xue et al. [21] the genipin-crosslinked donor sclera for posterior scleral reinforcement, findings in this study reveal favorable safety and effectiveness of this type of surgery to restrain eye globe elongation in patients < 18-years old within a 2- to 3-year follow-up.

Our results displayed significant heterogeneity in the pooled analysis of change in SE, AL, BCVA LogMAR, and AL/hCRC in PSR and control groups, the possible reasons may be attributed to study design, patients’ characteristics, procedure of surgery, experience of surgeons. In addition, 3 studies used self-control which may be another cause of high heterogeneity in this study. Subgroup analysis was not carried out due to the limited number of studies in each subgroup. All these potential sources for significant heterogeneity need to be further investigated. *Egger’s* tests for publication bias revealed that there was no significant publication bias in the current study. Results of sensitivity analysis suggested that the pooled outcomes in this study were robust after omitting study one after another. This study provided updated information on the effectiveness and safety of PSR the treatment of children with myopia. Of the eight included studies, seven were conducted in China. Therefore, we cannot be certain of the applicability of the results of this meta-analysis in other countries, and practitioners should interpret the results with care in the context of the Chinese healthcare system and patient characteristics when applying and interpreting the results. Moreover, subgroup analysis based on surgical technique was not done due to limited number of included studies, more relevant studies are needed to investigate the efficacy and safety under different surgical approaches.

## Conclusions

Despite the limitations of the current study, we may conclude that PSR is an effective operation to control the development of myopia and axial elongation, although adverse effects including edema and congestion are inevitable, they are durable and can be alleviated in several weeks. Well-designed studies with large sample sizes are warranted.

## Supplementary Information

Below is the link to the electronic supplementary material.

Supplementary file1 (TIFF 36 KB)

Supplementary file2 (TIFF 33 KB)

Supplementary file3 (TIFF 34 KB)

Supplementary file4 (TIFF 33 KB)

Supplementary file5 (DOCX 21 KB)

Supplementary file6 (DOCX 12 KB)

## Data Availability

The datasets used and/or analyzed during the current study are available from the corresponding author on reasonable request.
