# Supplementary figures and images for: Effect and safety posterior scleral reinforcement on controlling myopia in children: a meta-analysis
Source: Int Ophthalmol. 2024 Feb 6;44(1):8. doi: 10.1007/s10792-024-02929-w (PMC10847067; doi:10.1007/s10792-024-02929-w)

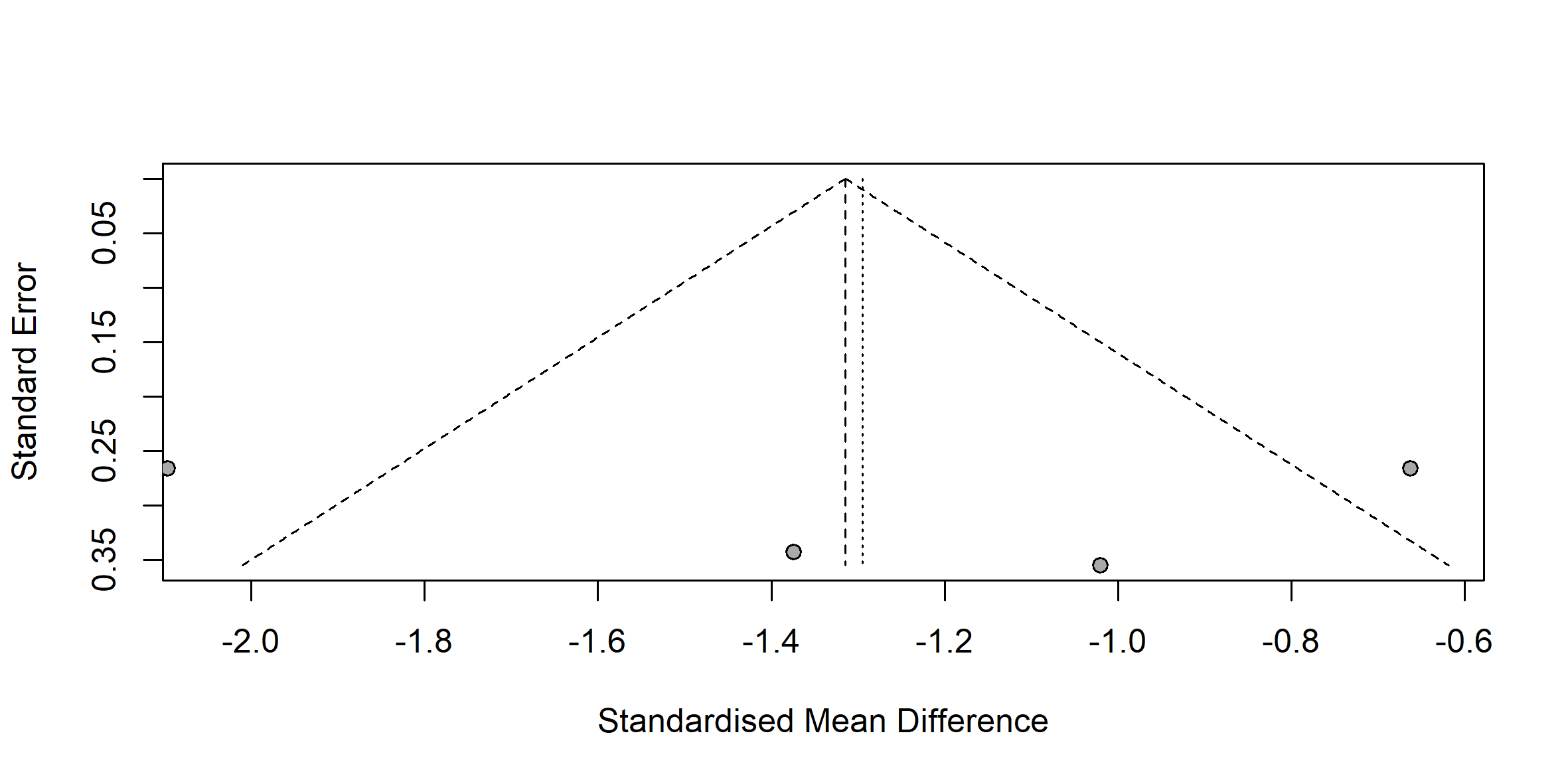

Supplement: Supplementary file 1 — Supplementary file1 (TIFF 36 KB) [file 10792_2024_2929_MOESM1_ESM.tiff]

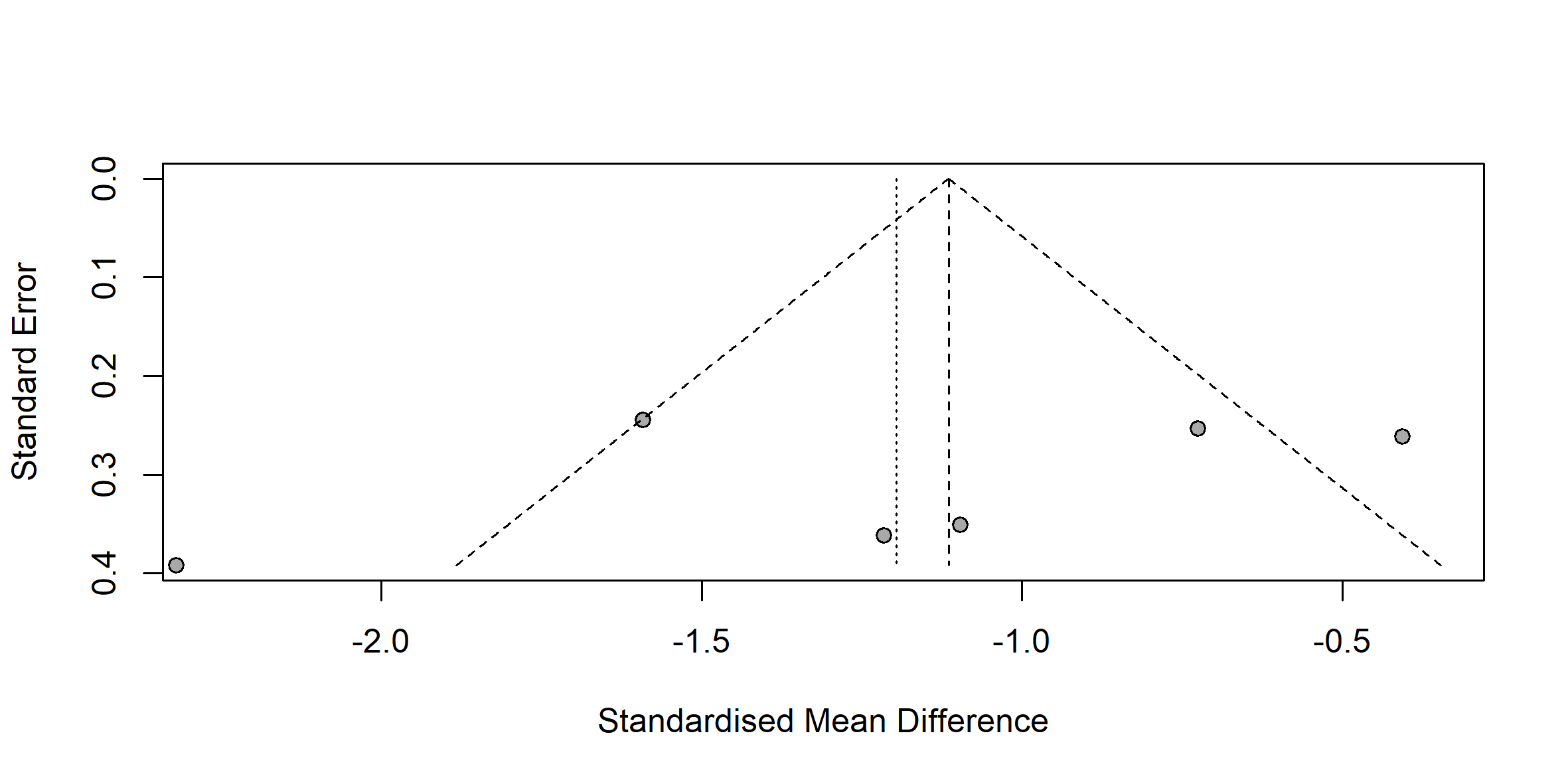

Supplement: Supplementary file 2 — Supplementary file2 (TIFF 33 KB) [file 10792_2024_2929_MOESM2_ESM.tiff]

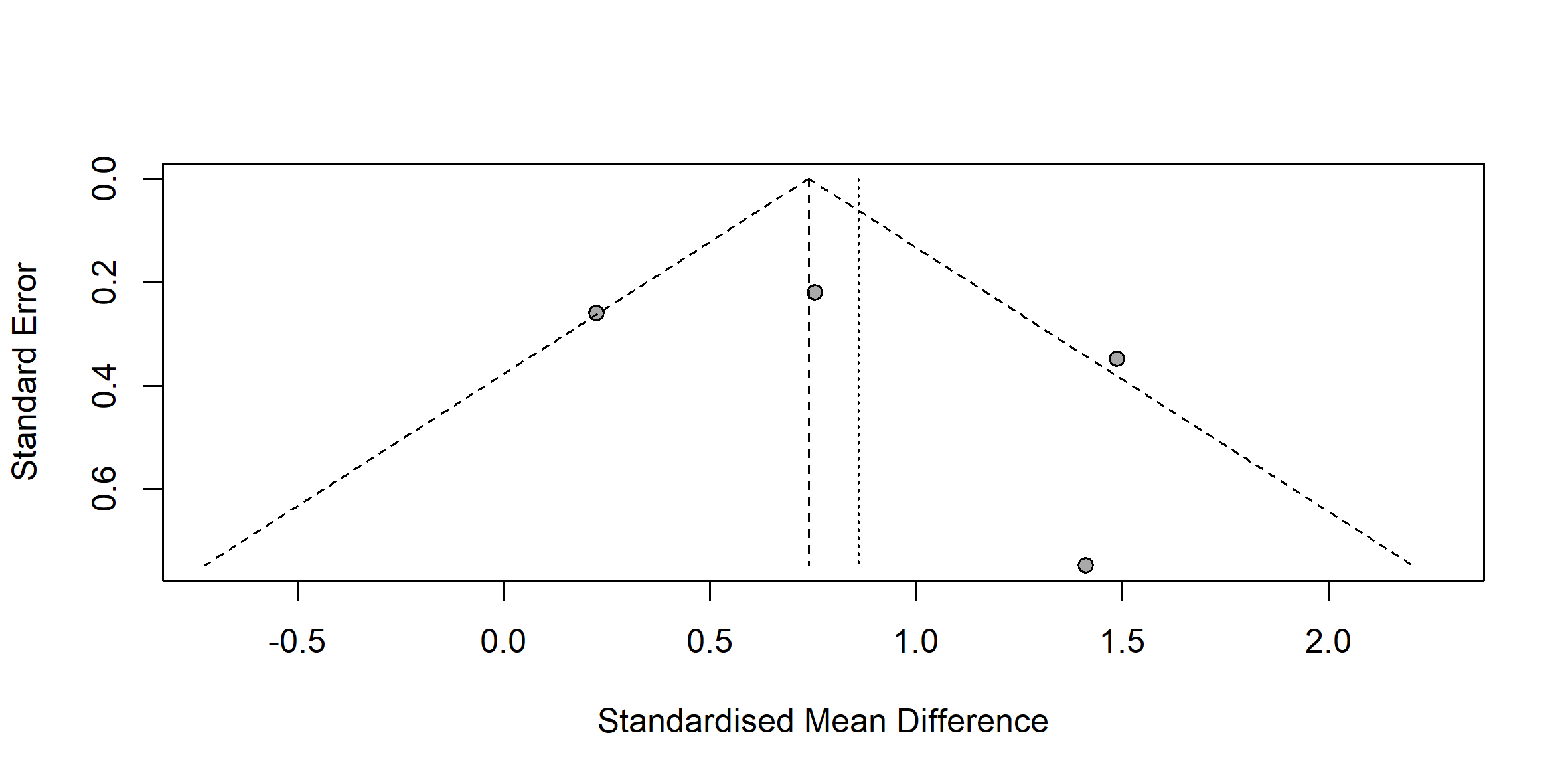

Supplement: Supplementary file 3 — Supplementary file3 (TIFF 34 KB) [file 10792_2024_2929_MOESM3_ESM.tiff]

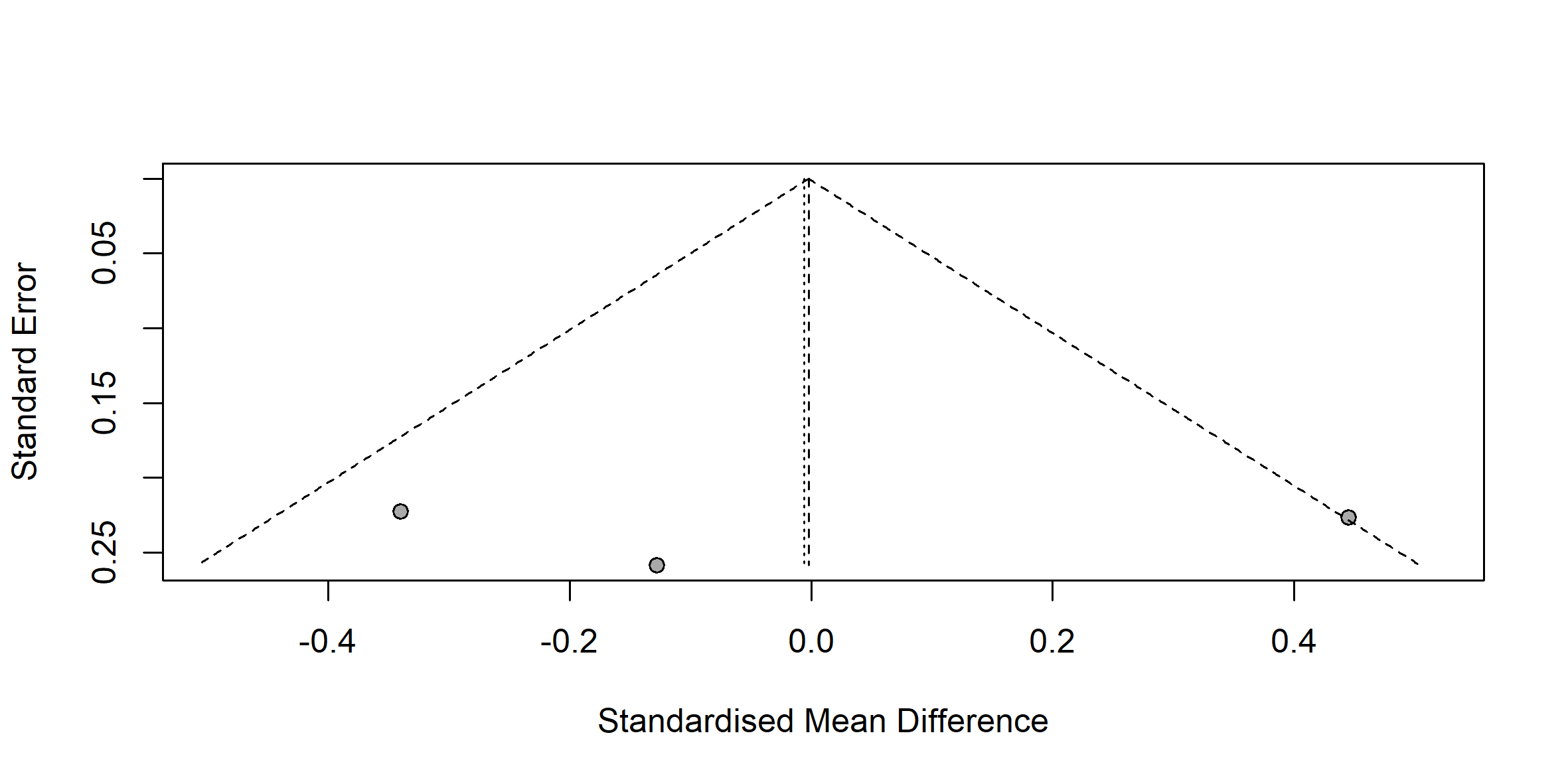

Supplement: Supplementary file 4 — Supplementary file4 (TIFF 33 KB) [file 10792_2024_2929_MOESM4_ESM.tiff]
