# Supplementary material for: Effect and safety posterior scleral reinforcement on controlling myopia in children: a meta-analysis
Source: Int Ophthalmol. 2024 Feb 6;44(1):8. doi: 10.1007/s10792-024-02929-w (PMC10847067; doi:10.1007/s10792-024-02929-w)
Supplement: Supplementary file 5 — Supplementary file5 (DOCX 21 KB) [file 10792_2024_2929_MOESM5_ESM.docx]

**Table S1 Search strategies for databases**

**PubMed**

| Search number | Query | Filters | Results |
| --- | --- | --- | --- |
| 9 | (myopia [mesh]) AND (((((scleral buckle [mesh]) OR (Posterior[tw] AND sclera*[tw] AND reinforce*[tw])) OR (buckl*[tw] AND reinforce*[tw])) OR (scleroplasty [mesh])) OR (snyder thompson)) | English | 199 |
| 8 | (myopia [mesh]) AND (((((scleral buckle [mesh]) OR (Posterior[tw] AND sclera*[tw] AND reinforce*[tw])) OR (buckl*[tw] AND reinforce*[tw])) OR (scleroplasty [mesh])) OR (snyder thompson)) | | 263 |
| 7 | ((((scleral buckle [mesh]) OR (Posterior[tw] AND sclera*[tw] AND reinforce*[tw])) OR (buckl*[tw] AND reinforce*[tw])) OR (scleroplasty [mesh])) OR (snyder thompson) | | 4,149 |
| 6 | snyder thompson | | 857 |
| 5 | scleroplasty [mesh] | | 81 |
| 4 | buckl*[tw] AND reinforce*[tw] | | 145 |
| 3 | Posterior[tw] AND sclera*[tw] AND reinforce*[tw] | | 70 |
| 2 | scleral buckle [mesh] | | 3,029 |
| 1 | myopia [mesh] | | 20,050 |

**Embase**

| No. | Query | Results |
| --- | --- | --- |
| #6 | #1 AND #4 AND [english]/lim | 158 |
| #5 | #1 AND #4 | 206 |
| #4 | #2 OR #3 | 846 |
| #3 | 'scleral buckle'/exp OR 'posterior scleral reinforcement' OR ('sclera*' NEAR/6 'reinforce*') OR ('buckl*' NEAR/6 'reinforce*') OR 'snyder thompson' | 828 |
| #2 | 'scleroplasty'/exp | 36 |
| #1 | 'myopia'/exp OR myopia | 36854 |

**Web of Science**

| Search number | Query | Results |
| --- | --- | --- |
| 8 | #6 AND #1 and English (Languages) | 25 |
| 7 | #6 AND #1 | 34 |
| 6 | #2 OR #3 OR #4 OR #5 | 931 |
| 5 | snyder thompson (Title) | 2 |
| 4 | scleroplasty (Title) | 23 |
| 3 | Posterior scleral reinforce* (Title) | 32 |
| 2 | Scleral Buckling (Title) | 875 |
| 1 | Myopia (Title) | 8193 |

**Cochrane Library**

| Search number | Query | Results |
| --- | --- | --- |
| 1 | MeSH descriptor: [Myopia] this term only | 1119 |
| 2 | (myopia):ti,ab,kw | 2834 |
| 3 | (posterior scleral reinforcement):ti,ab,kw | 3 |
| 4 | (snyder thompson):ti,ab,kw | 1 |
| 5 | (scleral buckle):ti,ab,kw | 235 |
| 6 | #1 OR #2 | 2834 |
| 7 | #3 OR #4 OR #5 | 239 |
| 8 | #6 AND #7 | 19 |
