# Supplementary material for: Effect and safety posterior scleral reinforcement on controlling myopia in children: a meta-analysis
Source: Int Ophthalmol. 2024 Feb 6;44(1):8. doi: 10.1007/s10792-024-02929-w (PMC10847067; doi:10.1007/s10792-024-02929-w)
Supplement: Supplementary file 6 — Supplementary file6 (DOCX 12 KB) [file 10792_2024_2929_MOESM6_ESM.docx]

**Fig. S1** Funnel plot of SMD for mean change in SE from the time of surgery to the end of the follow-up period in the PSR and control group

**Fig. S2** Funnel plot of SMD for the increase in AL in the PSR and control group

**Fig. S3** Funnel plot of SMD for decrease of BCVA in LogMAR in the PSR and control group

**Fig. S4** Funnel plot of SMD for change in (IOP) at the time of surgery and at the end of the follow-up period in the PSR group
